# Complete Chloroplast Genome of the Inverted Repeat-Lacking Species *Vicia bungei* and Development of Polymorphic Simple Sequence Repeat Markers

**DOI:** 10.3389/fpls.2022.891783

**Published:** 2022-05-16

**Authors:** Ick-Hyun Jo, Seahee Han, Donghwan Shim, Hojin Ryu, Tae Kyung Hyun, Yi Lee, Daeil Kim, Yoon-Sup So, Jong-Wook Chung

**Affiliations:** ^1^Department of Herbal Crop Research, National Institute of Horticultural and Herbal Science, Rural Development Administration, Eumseong, South Korea; ^2^Division of Botany, Honam National Institute of Biological Resources, Mokpo, South Korea; ^3^Department of Biological Sciences, Chungnam National University, Daejeon, South Korea; ^4^Department of Biology, Chungbuk National University, Cheongju, South Korea; ^5^Department of Industrial Plant Science and Technology, Chungbuk National University, Cheongju, South Korea; ^6^Department of Horticulture, Chungbuk National University, Cheongju, South Korea; ^7^Department of Crop Science, Chungbuk National University, Cheongju, South Korea

**Keywords:** chloroplast genome, inverted repeat-lacking clade, phylogeny, SSR marker, *Vicia bungei*

## Abstract

**Background:**

*Vicia bungei* is an economically important forage crop in South Korea and China. Although detailed genetic and genomic data can improve population genetic studies, conservation efforts, and improved breeding of crops, few such data are available for *Vicia* species in general and none at all for *V. bungei*. Therefore, the main objectives of this study were to sequence, assemble, and annotate *V. bungei* chloroplast genome and to identify simple sequence repeats (SSRs) as polymorphic genetic markers.

**Results:**

The whole-genome sequence of *V. bungei* was generated using an Illumina MiSeq platform. De novo assembly of complete chloroplast genome sequences was performed for the low-coverage sequence using CLC Genome Assembler with a 200–600-bp overlap size. *Vicia bungei* chloroplast genome was 130,796-bp long. The genome lacked an inverted repeat unit and thus resembled those of species in the inverted repeat-lacking clade within Fabaceae. Genome annotation using Dual OrganellarGenoMe Annotator (DOGMA) identified 107 genes, comprising 75 protein-coding, 28 transfer RNA, and 4 ribosomal RNA genes. In total, 432 SSRs were detected in *V. bungei* chloroplast genome, including 64 mononucleotides, 14 dinucleotides, 5 trinucleotides, 4 tetranucleotides, 233 pentanucleotides, 90 hexanucleotides, and 14 complex repeated motifs. These were used to develop 232 novel chloroplast SSR markers, 39 of which were chosen at random to test amplification and genetic diversity in *Vicia* species (20 accessions from seven species). The unweighted pair group method with arithmetic mean cluster analysis identified seven clusters at the interspecies level and intraspecific differences within clusters.

**Conclusion:**

The complete chloroplast genome sequence of *V. bungei* was determined. This reference genome should facilitate chloroplast resequencing and future searches for additional genetic markers using population samples. The novel chloroplast genome resources and SSR markers will greatly contribute to the conservation of the genus *Vicia* and facilitate genetic and evolutionary studies of this genus and of other higher plants.

## Introduction

*Vicia* L. is a genus in the Fabaceae family containing approximately 180–210 species. These species are widely distributed across temperate regions of the northern hemisphere and extend to temperate regions of South America and tropical Africa (Hanelt and Mettin, 1989). *Vicia* species are used as green manure, cover, forage, and honey crops, making it an economically important genus and a valuable genetic resource (Montemurro et al., 2013). Despite their high economic value, very few genetic and genomic data are available for species of *Vicia*, other than *Vicia villosa* (hairy vetch) and *Vicia faba* (broad bean). *Vicia bungei*, native to South Korea and China, is phenotypically and ecologically similar to *Vicia americana* (Endo et al., 2000). Although several chloroplast genomes of different *Vicia* species have been obtained through next-generation sequencing (NGS) (Cooper et al., 2017; Li et al., 2018; Xin and Yang, 2020), to date, no genetic or genomic studies have been conducted on *V. bungei*, despite the potentially valuable genetic resources present in wild varieties of this species. In addition, more effective molecular markers are required to support the phylogenetic and population genetic studies underlying the identification, conservation, utilization, and breeding of *Vicia* species.

The chloroplast genome has long been a focus of research into plant molecular evolution and systematics because of its small size, high copy number, and conservation among species. It has been extensively characterized at the molecular level (Kim et al., 2005). Recent technical advances in NGS technologies mean that the number of completely sequenced chloroplast genomes has increased rapidly, and such sequences play a progressively important role in the identification of molecular markers and in molecular phylogenetic analyses (Kim et al., 2016). Chloroplast genetic markers are potentially more effective indicators of population subdivision and differentiation than are nuclear markers (Schaal et al., 1998; Petit et al., 2005). Chloroplast simple sequence repeats (cpSSRs), generally defined as microsatellites with tandem repeats of 1–6 bp, are valuable resources for assessing genetic and genome diversity, as well as in phylogenetic and systematic evolutionary analyses; cpSSRs have several advantageous characteristics, including haploidy, non-recombination, uniparental inheritance, and low nucleotide substitution rate (Powell et al., 1995). The chloroplast genome can provide unique insight into evolutionary processes as it retains ancient genetic patterns (Provan et al., 2001). Moreover, as the genetic information in angiosperm chloroplasts is inherited maternally, chloroplast markers serve as useful indicators of maternal ancestry (Raveendar et al., 2015).

A new complete chloroplast genome sequence for *V. bungei* has been generated and compared with sequences of related genera in the Fabaceae family. This comparison enabled the development of cpSSR markers for future population genetics studies, explaining the structure. Our findings help explaining the structure of the complete *V. bungei* chloroplast genome and reveal evolutionary relationships within the genus *Vicia*.

## Materials and Methods

### Plant Material and DNA Extraction

Leaves from 1-year-old *V. bungei* plants were collected from the Industrial Plant Science and Technology greenhouse at Chungbuk National University (Cheongju, South Korea; 36° 37′ 44.3″ N, 127° 27′ 02.5″ E), immediately snap frozen in liquid nitrogen, and stored at −80°C until analysis. DNA was extracted using automated QIACube system (Qiagen, Hilden, Germany) with a DNeasy Plant Mini Kit (Qiagen, Hilden, Germany) according to the manufacturer’s protocol. Qualitative and quantitative assessment of the DNA samples was performed by spectrophotometry (NanoDrop; Thermofisher, Waltham, MA, United States) before their use in the DNA Sequencing.

### Sequencing, Assembly, Phylogenetic Relationships, and Comparison of Chloroplast Genome

DNA Sequencing was conducted using the Illumina MiSeq sequencing platform (Illumina, San Diego, CA, United States), and 2.0 Gb of sequence data was generated. Quality trimming and assembly of the reads were achieved using the dnaLCW method (Kim et al., 2015) and CLC Assembly Cell version 4.21 (CLC Inc., Qiagen, Aarhus, Denmark). Phylogenetic relationships were analyzed with the maximum-likelihood method using 61 conserved chloroplast protein sequences from 20 Fabaceae species (downloaded from GenBank; see Supplementary Figure 1) and *V. bungei*. The analysis was conducted in MEGA7 (Kumar et al., 2016) with 1,000 bootstrap replicates. The complete chloroplast genome of *V. bungei* was compared with five published chloroplast genomes (*Vicia sativa, V. faba, Vicia sepium, Vicia ramuliflora, and Vicia costata*) using the mVISTA program (Mayor et al., 2000).

### Chloroplast Simple Sequence Repeat Detection and Primer Design

Simple sequence repeat mining was performed using the MIcroSAtellite identification tool (Thiel et al., 2003). The following search parameters were set for identification: mono-, di-, tri-, tetra-, penta-, and hexa-nucleotide motifs with a minimum of ten, five, four, three, two, and two repeats, respectively. The primers for SSR markers were designed using Primer 3.0 software.^1^ The parameters for designing the primers were set as follows: a primer length of 18–22 bp, with 20 bp set as the optimum value; an optimum annealing temperature of 58°C; and a polymerase chain reaction (PCR) product size of 100–300 bp.

### Chloroplast Simple Sequence Repeat Marker Validation and Data Analysis

A total of 39 developed CpSSR markers were randomly selected to assess the genetic diversity of *Vicia* species (20 accessions from seven species; Supplementary Table 1). The PCR mixture (total volume 40 μl) contained 20 ng genomic DNA, 10 pmol each primer, 2.5 mM MgCl_2_, 0.25 mM dNTPs, and 0.5 U Taq polymerase (Inclone, Deajeon, South Korea). Polymerase chain reaction amplification was performed under the following conditions: 94°C for 1 min; 30 cycles of 94°C for 30 s, 55°C for 30 s, and 72°C for 30 s; and a final extension at 72°C for 5 min. The size of PCR products was analyzed using the Fragment Analyzer (Advanced Analytical Technologies Inc., Ankeny, IA, United States), and allele sizes were scored using the PROSize 2.0 (Advanced Analytical Technologies). The number of alleles, the major allele frequency, the expected heterozygosity and polymorphic information content were calculated using the PowerMarker v3.25.^2^

The expected heterozygosity formula is as follows:


$$\hat{D_{l}}=\left(1-\sum_{u=1}^{k}{\tilde{p}}_{l\,u}^{2}\right).$$

A closely related diversity measure is the polymorphism information content (*PIC*):


$$\hat{P\,I\,C_{l}}=1-\sum_{u=1}^{k}{\tilde{p}}_{l\,u}^{2}-\sum_{u-1}^{k-1}\sum_{v=u+1}^{k}2\,{\tilde{p}}_{l\,u}^{2}\,{\tilde{p}}_{l\,v}^{2}.$$

Phylogenetic analysis of *Vicia* species (20 accessions from seven species) was performed using UPGMA cluster analysis, and unrooted tree construction was based on the CS chord 1967 distance method in PowerMarker v3.25 software.

## Results

### Genomic Characteristics and Phylogenetic Relationships With Fabaceae

**FIGURE 1:**
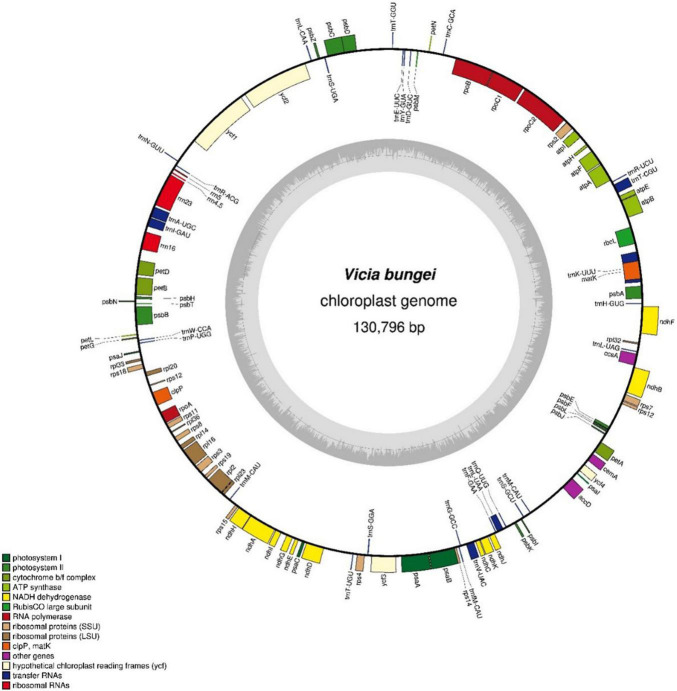
Schematic map of the chloroplast genome of *Vicia bungei*.

The complete chloroplast genome of *V. bungei* was 130,796-bp long and lacked an inverted repeat unit (Figure 1). The overall GC content was 34.73%. A total of 107 genes were identified, including 75 protein-coding, 28 transfer RNA, and 4 ribosomal RNA genes (DNA-directed RNA polymerase genes) (Supplementary Table 2). Furthermore, 18 ribosomal subunit genes (ten small subunits and eight large subunits) were detected. Eleven genes, including *petB*, *petD*, *atpF*, *ndhA*, *ndhB*, *rpl16*, *rpl2*, *rps12*, *rpoC1*, *clpP*, and *ycf3*, contained one or two introns. Additionally, *rps12* was identified as a *trans*-splicing gene.

A total of 45 chloroplast genes were detected and involved in photosynthesis, and encoded subunits of NADH oxidoreductase (11 genes), subunits of photosystem I (seven genes), subunits of photosystem II (14 genes), subunits of the cytochrome b6/f complex (seven genes), different subunits of ATP synthase (seven genes), and the large chain of ribulose bisphosphate carboxylase (one gene). In addition, five genes were involved in different functions, which two of them remained unknown (Table 1). *Vicia bungei* chloroplast genome resembled to other *Vicia* species plastomes in the inverted repeat-lacking clade (IRLC), as it lacked *rpl22*, *rps16*, and one intron of *clpP* (Kim et al., 2016; Li et al., 2018; Xin and Yang, 2020). The complete chloroplast genome sequence, with gene annotations, was submitted to GenBank (accession number MT362055).

**TABLE 1:** Genes present in the *Vicia bungei* chloroplast genome.

Role	Gene
Photosystem I	*psaA, psaB, psaC, psaI, psaJ, ycf3*^b^*, ycf4*
Photosystem II	*psbB, psbC, psbD, psbE, psbF, psbH, psbI, psbJ, psbK, psbL, psbM, psbN, psbT, psbZ*
Cytochrome b6/f	*petA, petB*^a^*, petD*^a^*, petG, petL, petN*
ATP synthase	*atpA, atpB, atpE, atpF*^a^*, atpH, atpI*
Rubisco	*rbcL*
NADH oxidoreductase	*ndhA*^a^*, ndhB*^a^*, ndhC, ndhD, ndhE, ndhF, ndhG, ndhH, ndhI, ndhJ, ndhK*
Large subunit ribosomal proteins	*rpl14, rpl16*^a^*, rpl2*^a^*, rpl20, rpl23, rpl32, rpl33, rpl36*
Small subunit ribosomal proteins	*rps11, rps12*^a^^,^^c^*, rps15, rps18, rps19, rps2, rps3, rps4, rps7, rps8*
RNA polymerase	*rpoA, rpoB, rpoC1*^a^*, rpoC2*
Unknown function protein-coding gene	*ycf1, ycf2*
Other gene	*accD, ccsA, cemA, clpP*^a^*, matK*
Ribosomal RNAs	*rrn16, rrn23, rrn4.5, rrn5*
Transfer RNAs	*trnA-UGC*^a^*, trnC-GCA, trnD-GUC, trnE-UUC, trnF-GAA, trnG-GCC, trnH-GUG, trnI-GAU*^a^*, trnK-UUU*^a^*, trnL-CAA, trnL-UAA*^a^*, trnL-UAG, trnM-CAU, trnN-GUU, trnP-UGG, trnQ-UUG, trnR-ACG, trnR-UCU, trnS-GCU,trnS-GGA, trnS-UGA, trnT-CGU*^a^*, trnT-GGU, trnT-UGU, trnV-UAC*^a^*, trnW-CCA, trnY-GUA, trnfM-CAU*

*^a^Gene containing a single intron.*

*^b^Gene containing two introns.*

*^c^Trans-splicing gene.*

Phylogenetic analysis for 21 Fabaceae species, based on 61 conserved plastid protein sequences, showed that *V. bungei* clustered with two species from the same genus, *V. sepium* and *V. sativa*, supported by high bootstrap values in the maximum-likelihood tree (Supplementary Figure 1).

### Development of Chloroplast Simple Sequence Repeat Markers

A total of 432 simple sequence repeats (SSRs) were detected, which included 64 mononucleotides, 14 dinucleotides, 5 trinucleotides, 4 tetranucleotides, 233 pentanucleotides, 98 hexanucleotides, and 14 complex repeated motifs, in the chloroplast genome of *V. bungei* (Supplementary Figure 2). The majority of the identified SSRs were pentanucleotide repeats (53.9%), followed by hexanucleotides repeats (22.7%). A total of 432 potential SSR motifs were identified, 313 (73.45%) of which occurred within the intergenic regions (Supplementary Table 3).

A total of 232 pairs of SSR primers were designed from 432 potential SSR motifs (Supplementary Table 4) that showed potential for marker development; these included mono-, di-, tetra-, penta-, and hexanucleotides, and complex repeated motifs. Pentanucleotides (54.31%) were the most abundant group within the selected SSR markers, followed by hexa- (25%), mono- (14.65%), di- (6%), and tetranucleotides (4%) and complex repeated nucleotide (4%) markers (Supplementary Table 4). The most common motif from the pentanucleotide markers was the GAATT/GAAAT (4.76%), followed by CAAAA/CATAA (3.97%), AAAGA/AATGA (3.97%), and TATAT/TATTT (3.17%) (Supplementary Table 4).

### Genetic Diversity in *Vicia* Species

Of the 232 cpSSR markers described above, 39 were selected randomly, in order to evaluate their amplification potential and to assess genetic diversity in the genus *Vicia* (20 accessions from seven species). Amplification of all the selected cpSSR markers produced clear fragments; 35 fragments showed polymorphisms, and four were monomorphic. The major allele frequencies within the 39 SSR markers across 20 accessions (*Vicia* spp.) ranged from 0.20 (VBCP38 and VBCP42) to 1.0 (VBCP41, VBC54, VBC65, and VBC164), with a mean value of 0.62. The number of alleles per marker varied between 1 (VBCP41, VBC54, VBC65, and VBC164) and 16 (VBCP42), with a mean value of 3.92. The expected heterozygosity ranged from 0 (VBCP41, VBC54, VBC65, and VBC164) to 0.92 (VBCP39), with a mean value of 0.48. The polymorphic information content ranged from 0 (VBCP41, VBC54, VBC65, and VBC164) to 0.91 (VBCP39), with a mean value of 0.569 (Table 2). The most polymorphic loci (i.e., those exhibiting the highest diversity) were VBCP39 and VBCP42, based on their relatively large allele number (15 and 16, respectively).

**TABLE 2:** Summary of the 39 polymorphic chloroplast simple sequence repeat markers and their genetic diversity statistics across 20 *Vicia* spp. accessions.

Marker	Location	Repeat motif	Left sequence	Right sequence	M_AF_	N_A_	H_E_	PIC
VBCP35	rpoB	(AT)n	TATCAACGGGTCTTCCATCTTG	CGAGCTATACTTGGGATTCAGG	0.50	4.00	0.67	0.62
VBCP37	rpoC2	(TA)n	ATAACACAATCGGCAGATCCAA	TTCTGTAAACACCCGAAATGGA	0.70	4.00	0.48	0.44
VBCP38	trnK-UUU∼matK	(TA)n	CACGGCTTTCCCTATGTATACA	TGCAGAGGTTCCATAGAAATCG	0.20	11.00	0.89	0.87
VBCP39	psbB∼petL	(TA)n	TCAGTGAATACAGACAATGGATAC	ATCATCTGTGACATCTACGCAG	0.16	15.00	0.92	0.91
VBCP40	ycf1	(TC)n	ATAGAATGCTCTCCCAAGCTTC	TTTCGAGATACTGGGCGACTA	0.43	4.00	0.66	0.59
VBCP41	psbB	(AACC)n	AAAGGAACATCCGCTCTAACAA	ACATCGGTAATAATCCGGCAAA	1.00	1.00	0.00	0.00
VBCP42	rpl32∼ndhF	(ATTA)n	TCCTCATCCTCGCTCTATAGAT	TCTTTGCACAATGGTCCCAATA	0.20	16.00	0.90	0.90
VBCP43	ndhD	(TATT)n	ATCAATGGCTTCTCTTGCATTG	CGAAATAAATAATTCTCTGGGCCC	0.80	2.00	0.32	0.27
VBCP44	psaB	(TTTC)n	CGGTGTTTATCAGTGGTGGTAT	CAAGCTAAGGAACTGACTCCAA	0.80	2.00	0.32	0.27
VBCP53	rpoB∼trnC-GCA	(AACAA)n	TCATTCTTCATCGAATCACATGA	ACCCGAAGTCTAGGTGAAATTT	0.75	3.00	0.41	0.37
VBCP54	psbD	(AACCC)n	TTCATATGATGGGAGTTGCTGG	GAGCACTCATCCATAAACCAGT	1.00	1.00	0.00	0.00
VBCP56	atpB	(AACTT)n	CCCAGGGAAATATGTTGGTCTA	CTTTCACTTCTGAATCCCAAACA	0.85	2.00	0.26	0.22
VBCP63	trnE-UUC∼trnT-GGU	(AATGA)n	AAGAATTGAGTTGAGGGACAGG	ACATAGCAACTCATTAACGAACA	0.45	5.00	0.68	0.63
VBCP65	rpoC2	(AATGG)n	TGAACTGTTATAACTTGACCCGA	TGGCAACTTGACAAATTAACTGA	1.00	1.00	0.00	0.00
VBCP69	psbM∼trnD-GUC	(ACCAA)n	GGATCTCGATGATATCAAATCGGA	AGATCATTTCGAACAGGTATCCC	0.60	3.00	0.56	0.49
VBCP75	rpoC1	(ATAAA)n	CCCTACTGTTTCTCCATTAGGT	TTTGGCTCTGGAACTGAATCAT	0.80	2.00	0.32	0.27
VBCP79	trnE-UUC∼trnT-GGU	(ATGAT)n	GAGATGTCCTAAACCGCTAGAC	AGATTGGTGATTGGAATGAACAA	0.85	3.00	0.27	0.25
VBCP81	atpH∼atpI	(ATTCA)n	TTTCGTTTCTACCCTTGTAGTTT	ACGGTATGGAACAAACACATGT	0.40	4.00	0.71	0.65
VBCP85	rbcL∼atpB	(ATTTG)n	CAAGAACAAGGTCTACTCGACA	TCACTGTCAAGGTCAAGAGTCT	0.45	4.00	0.63	0.55
VBCP88	rpoC2	(ATTTT)n	TTGGTGGAATAATGACGTTATGT	TGGGAGAAGCTGTAGGGATTAT	0.75	2.00	0.38	0.30
VBCP90	rpoC2	(CAAAA)n	TTGACAACTTTGAGTTCCAGATT	ACATAGTGCCATCTTGATACCG	0.70	4.00	0.48	0.44
VBCP98	trnK-UUU∼matK	(CATAA)n	CACGGCTTTCCCTATGTATACA	TGCAGAGGTTCCATAGAAATCG	0.25	6.00	0.82	0.79
VBCP99	matK∼trnK-UUU	(CATAA)n	CCTCGCTTCTTCCTTCTCATTT	CGATTAGTGCTTGCTGTGGAAA	0.73	4.00	0.43	0.39
VBCP100	petN∼psbM	(CATTG)n	CTGCTGGTTGTAGTCTGATCAT	TCGCATTTATAGCTACTGCACT	0.60	2.00	0.48	0.36
VBCP106	psbC	(CTTAT)n	ACATGTATGGTTGGGTTCCATT	AGTAAATGCTTGAGCTTGAGAAG	0.85	2.00	0.26	0.22
VBCP108	atpI∼rps2	(CTTTT)n	TGACCTACTTCCACAGCAGATA	TTTAGATTTGGTTGGGCGGG	0.80	3.00	0.34	0.30
VBCP109	rpoC2	(CTTTT)n	CGTTCTTGAATCGATTGGAATGG	ACTTCGCAAGGATCAAGATCAA	0.45	4.00	0.63	0.55
VBCP112	trnT-GGU∼psbD	(GAAAT)n	TCCTTTCATTGTCAGATACTCCT	AGATTCTTGCAGAGTGAGAACC	0.56	3.00	0.59	0.52
VBCP116	rpoC1∼rpoC1	(GAATG)n	AATTGACCATAGACCCATTCCC	CCTAGTTATATCGCGAGCCTTT	0.85	2.00	0.26	0.22
VBCP122	atpF	(GCACT)n	TTAGTAAGAAGTCATTCGCCGG	CTATCCATAAGAGGAGATGCGC	0.55	2.00	0.50	0.37
VBCP123	matK	(GGATA)n	CCAATTACAAAGAAACAGCCGT	TCTTCCTTAGAGGAGGCAGAAA	0.90	2.00	0.18	0.16
VBCP127	trnD-GUC∼trnY-GUA	(GTATA)n	GACTCGAACCCGCAACTTCC	CGAGTCATCCGTGTCGATAAAG	0.50	5.00	0.69	0.65
VBCP131	trnY-GUA∼trnE-UUC	(TACCC)n	ATTGCCAACGAATTTACAGTCC	CATAGTAGAATGGAAGTCGGGC	0.80	2.00	0.32	0.27
VBCP144	rpoC1∼rpoC1	(TCTAA)n	TCCTCTCATCCGGCTAAAGTAT	TTTCTGTCGTAATTTCGAATTGCA	0.55	3.00	0.57	0.48
VBCP149	rpoC2	(TGATT)n	GGGACATTAGTTCGTTCTTTCG	ACCATGGATTCACTTTCTAATGGA	0.35	5.00	0.77	0.73
VBCP155	petN∼psbM	(TTATT)n	GGGAAGAAGTGGACTCTAAAGG	GGCAACAATTTCAATATTTGTGTG	0.25	5.00	0.77	0.73
VBCP156	trnE-UUC∼trnT-GGU	(TTCAA)n	AAGAATTGAGTTGAGGGACAGG	ACATAGCAACTCATTAACGAACA	0.30	7.00	0.79	0.76
VBCP162	trnE-UUC∼trnT-GGU	(TTGAG)n	TTGTATTTCACACTAAGTCGGAAA	ACCGATTTGAATTGAAGTCATCT	0.65	2.00	0.46	0.35
VBCP164	atpE∼trnT-CGU	(TTTAG)n	TAGGACACGAGTAGAGGCTATC	GTTCACATGTTTCGTAAAGGGC	1.00	1.00	0.00	0.00
Mean					0.62	3.92	0.48	0.43

**FIGURE 2:**
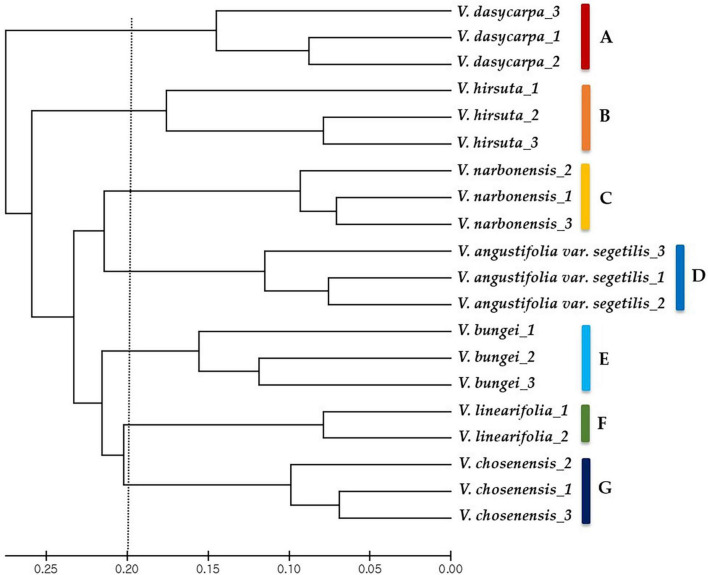
Phylogenetic relationships between 20 *Vicia* spp. accessions. The phylogenetic tree was constructed with data from 39 chloroplast simple sequence repeat markers using the unweighted pair group method with arithmetic mean method. Letters A–E on the right-hand side represent each plastome group based on the plastome haplotypes.

These 39 cpSSR markers were used to further analyze the genetic diversity of the 20 *Vicia* spp. accessions. The unweighted pair group method with arithmetic mean (UPGMA) cluster phylogenetic analysis clearly distinguished the accessions by genotype and grouped them into seven major clusters that corresponded with the different species (Figure 2). Targeted analysis of cpSSR regions in *V. bungei* identified a unique chloroplast type for each of the seven species examined (group A: *Vicia dasycarpa*; group B: *Vicia hirsuta*; group C: *Vicia narbonensis*; group D: *Vicia angustifolia* var. *segetilis*; group E: *V. bungei*; group F: *Vicia linearifolia*; group G: *Vicia chosenensis*). The UPGMA cluster analysis also revealed intraspecific variation between the 20 *Vicia* spp. accessions.

## Discussion

Most chloroplast genomes have a circular structure that contains a large single-copy region, an inverted repeat A region, a small single-copy region, and an inverted repeat B region (Raveendar et al., 2015; Zhu et al., 2016). The chloroplast genomes of some legumes, including *V. sepium*, have lost one of the two inverted repeats, and these species form the IRLC (Li et al., 2018). The chloroplast genome of *V. bungei* indicates that this economically important crop is also a member of the IRLC, suggesting that it may be part of the same evolutionary clade as *V. sepium* and *V. sativa*, which may share a similar evolutionary history.

A chloroplast gene is rarely lost arbitrarily. Instead, the gene is either transferred to the nuclear genome, or its function is replaced by a nuclear gene (Wang et al., 2018). Recent sequencing and analyses of some IRLC plastomes (Kim et al., 2005; Li et al., 2018; Moghaddam et al., 2022) have revealed important evolutionary patterns in this clade, including loss of the genes *rpl22* and *rps16*, the deletion of one intron of *clpP* (Jansen et al., 2008; Li et al., 2020), multiple sequence inversions (Schwarz et al., 2015; Williams et al., 2015), and gene transfers to the nucleus (Moghaddam and Kazempour-Osaloo, 2020; Wu et al., 2021). *Vicia bungei* plastome lacked one intron of *clpP*, which was consistent with the finding of Jansen et al. (2008) and confirmed the parallel loss of this *clpP* intron in *V. bungei* and in members of the papilionoid IRLC. Recently, Williams et al. (2015) demonstrated that the rates of synonymous and non-synonymous mutations are accelerated in the *clpP* sequence of *Acacia*, suggesting there may be a functional nuclear-encoded copy of this gene in at least some mimosoid legumes. The mechanism by which *rpl22*, *rps16*, and one intron of *clpP* gene have been lost from *V. bungei* requires further in-depth research.

Although the structure of plastid genomes is largely conserved across land plants (Wicke et al., 2011), other exceptions to this pattern, beyond these IRLC legumes, include Geraniaceae (Guisinger et al., 2011) and Campanulaceae (Haberle et al., 2008). Some Fabaceae plastomes have been highly rearranged owing to multiple rounds of translocations and/or inversions. As a result, the plastomes of the IRLC have undergone considerable diversification in both gene order and gene/intron content (Wang et al., 2018; Li et al., 2020; Xin and Yang, 2020; Moghaddam et al., 2022).

The plastome of *V. bungei* most closely resembled those of *V. sepium* and *V. sativa*. This finding is consistent with previous research that indicated similar evolutionary evaluation analysis of *V. sepium* and *V. sativa* (Li et al., 2020). Plastome differences between some major clades provide valuable information for resolving phylogenetic relationships based on DNA sequence analyses. The study of genomic variation and phylogeny of Fabaceae species provides an increased understanding of general chloroplast genome evolution. In our results of multiple alignments among six *Vicia* species cp genomes, we observed 11 major variant regions of *V. bungei* in cp genome (Supplementary Figure 3). However, our research focus on the development of cpSSR markers for *Vicia* species identification. Thus, these variant regions would be very useful for further evolutionary studies as well as cpSSR marker design.

In other flowering plant chloroplast genomes, the most common SSR motifs are mono-, di-, and trinucleotide repeats (George et al., 2015). Nevertheless, data mining, under the recursive criteria adopted here, revealed that the majority of SSRs present in the *V. bungei* chloroplast genome sequences contained relatively long penta- (53.9%) or hexanucleotide (22.7%) motifs. In this study, a total of 432 potential SSR were found in the chloroplast genome of *V. bungei*. The number of repeat motifs was richer in *V. bungei* chloroplast genome compared with the *V. sepium* chloroplast genome (Li et al., 2020). These results provide a firm foundation for further investigations of chloroplast genome evolution in *Vicia* L. and other IRLC legumes such as *Medicago* and *Pisum* and species.

Raveendar et al. (2015) used SSR markers to detect genetic diversity in *V. sativa* and 22 other *Vicia* species. Li et al. (2018) used chloroplast genome sequences to determine the genetic diversity in *V. sepium* and 21 closely related Fabaceae species. Han et al. (2021) used barcoding loci (ITS2, *matK*, and *rbcL*) as DNA markers to differentiate 19 *Vicia* taxa. Previous studies of phylogenetic relationships among species of the subgenus *Vicia* did not detect any intraspecific variation when cDNA SSR, cpSSR, or DNA barcoding markers were applied to representative plants of each species. By contrast, our UPGMA cluster analysis classified seven clusters at the interspecies level and revealed intraspecific differences within clusters. These results suggest that the 39 cpSSR markers developed in *V. bungei* differentiated efficiently between *Vicia* species genotypes and also provide estimates of their genetic diversity. These cpSSR markers will thus be useful, not only for authenticating *Vicia* species but also for providing the baseline data essential for advancing systematic breeding in the field and the development of conservation strategies, as well as for guiding the collection of germplasm.

## Conclusion

Complete chloroplast genomes have helped to reveal intraspecies relationships, but also allow to measure divergence within interspecies. Growing genomic resources for *Vicia* spp. provide tools to extend our knowledge on this critically important forage crop species. To develop cpSSR markers that can be utilized to classify *Vicia* species and analyze the genetic diversity of related species, potential cpSSR motifs were mined from the chloroplast genome of *V. bungei*, and finally, 39 cpSSR markers were developed. The UPGMA cluster analysis detected intra-and interspecific variation between 20 accessions with 39 cpSSR markers to distinguish *Vicia* species. The chloroplast genome and cpSSR markers found in this study would provide useful information for genetic diversity analyses in *Vicia* species.

## Data Availability Statement

The datasets presented in this study can be found in online repositories. The names of the repository/repositories and accession number(s) can be found in the article/Supplementary Material.

## Author Contributions

I-HJ and SH performed the experiments, analyzed the data, prepared figures and tables, and wrote the manuscript. DS, HR, TH, YL, DK, Y-SS, and J-WC reviewed drafts of the manuscript. All authors have read and approved the final manuscript.

## Conflict of Interest

The authors declare that the research was conducted in the absence of any commercial or financial relationships that could be construed as a potential conflict of interest.

## Publisher’s Note

All claims expressed in this article are solely those of the authors and do not necessarily represent those of their affiliated organizations, or those of the publisher, the editors and the reviewers. Any product that may be evaluated in this article, or claim that may be made by its manufacturer, is not guaranteed or endorsed by the publisher.

## Footnotes

1
http://frodo.wi.mit.edu/

2
https://brcwebportal.cos.ncsu.edu/powermarker/
