# Supplementary figures and images for: Complete Chloroplast Genome of the Inverted Repeat-Lacking Species Vicia bungei and Development of Polymorphic Simple Sequence Repeat Markers
Source: Front Plant Sci. 2022 May 16;13:891783. doi: 10.3389/fpls.2022.891783 (PMC9149428; doi:10.3389/fpls.2022.891783)

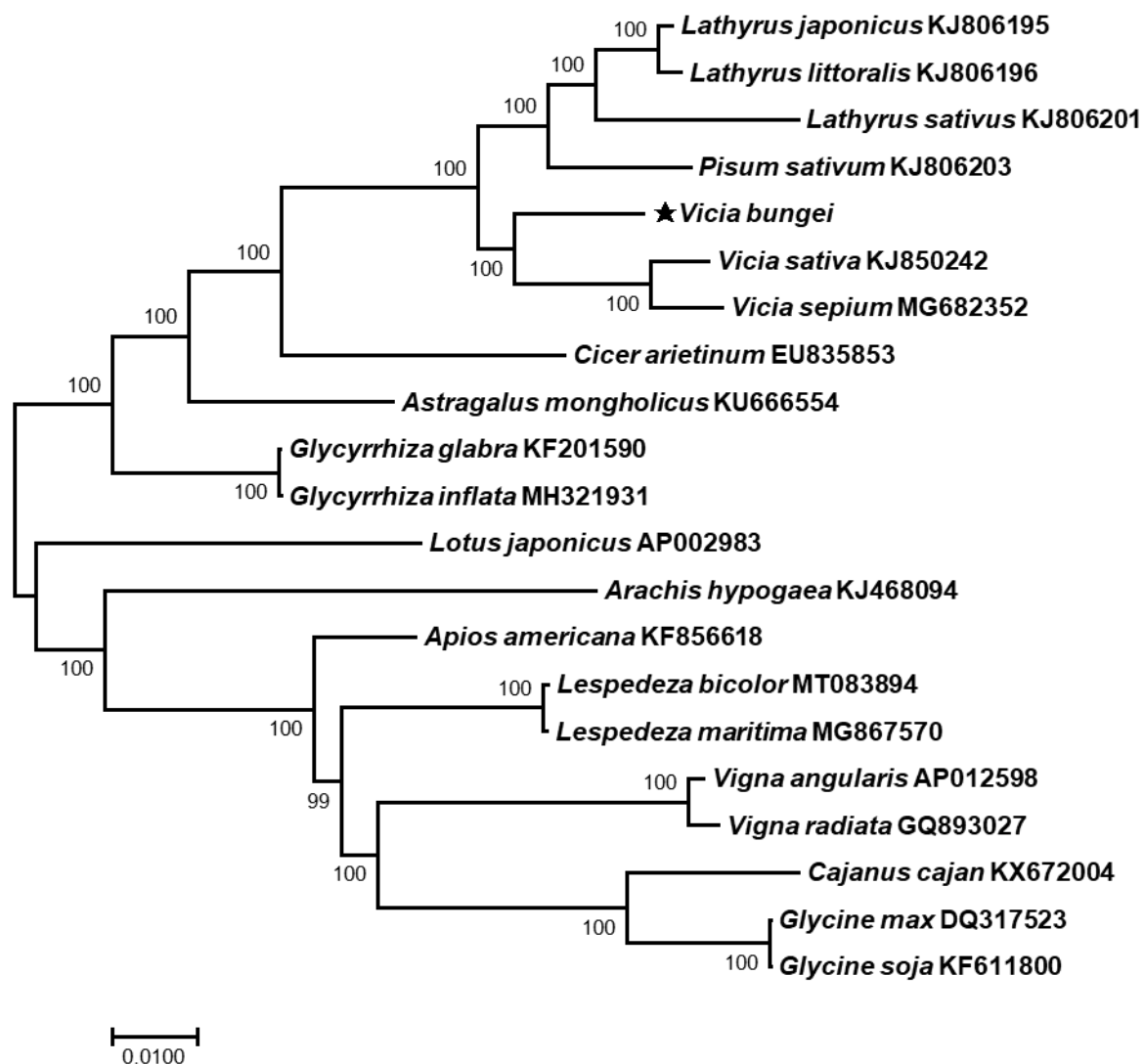

Fig. S1. Maximum-likelihood tree of *Vicia bungei* and 20 other Fabaceae species

Supplement: Supplementary file 1 [file Image_1.pdf]
