# Supplementary material for: Complete Chloroplast Genome of the Inverted Repeat-Lacking Species Vicia bungei and Development of Polymorphic Simple Sequence Repeat Markers
Source: Front Plant Sci. 2022 May 16;13:891783. doi: 10.3389/fpls.2022.891783 (PMC9149428; doi:10.3389/fpls.2022.891783)
Supplement: Supplementary file 2 [file Image_2.pdf]

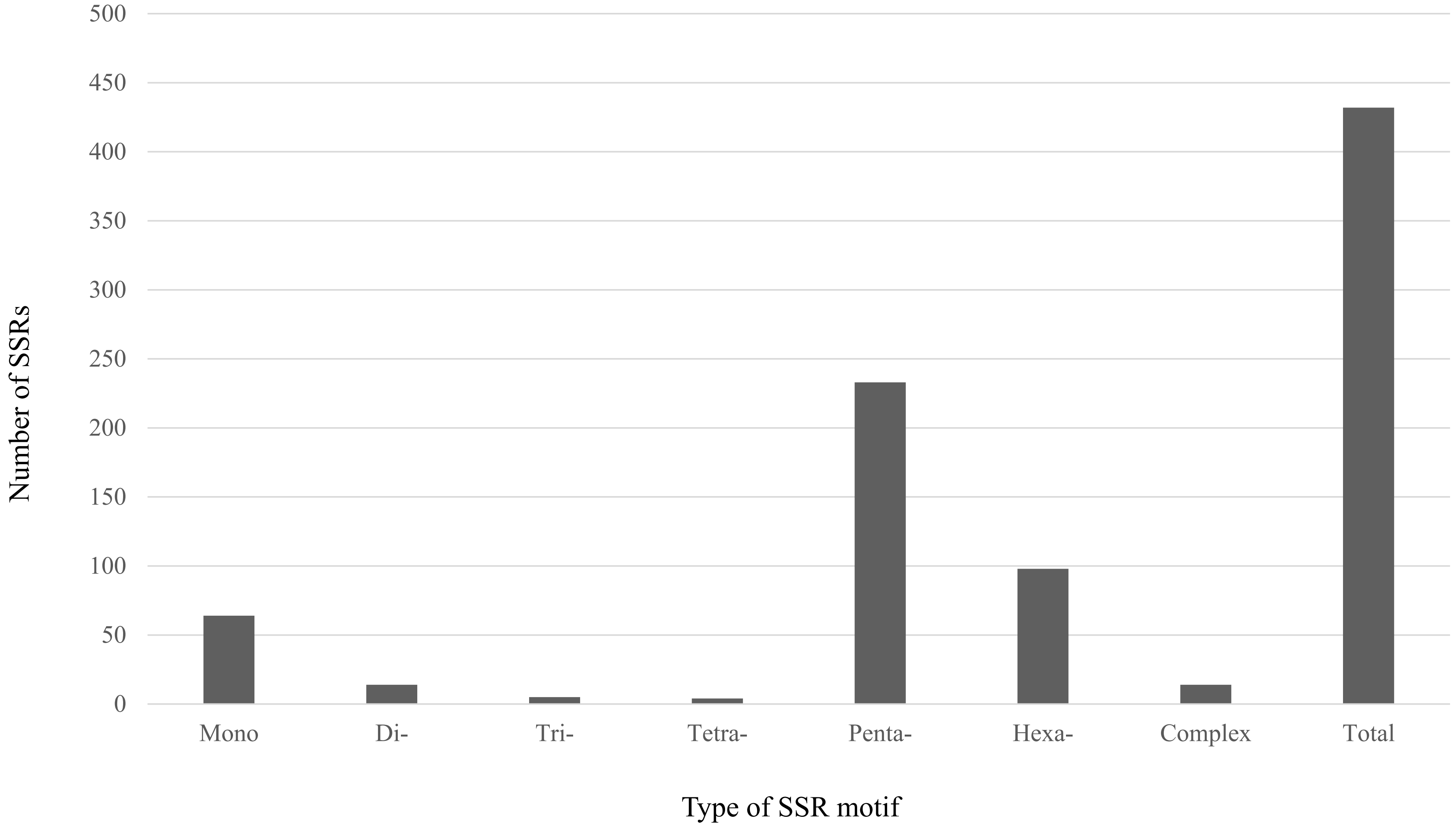

Fig. S2. The distributions and frequencies of SSR motifs with different repeat lengths identified in the *Vicia bungei* chloroplast genome
