# Supplementary material for: Complete Chloroplast Genome of the Inverted Repeat-Lacking Species Vicia bungei and Development of Polymorphic Simple Sequence Repeat Markers
Source: Front Plant Sci. 2022 May 16;13:891783. doi: 10.3389/fpls.2022.891783 (PMC9149428; doi:10.3389/fpls.2022.891783)
Supplement: Supplementary file 3 [file Image_3.pdf]

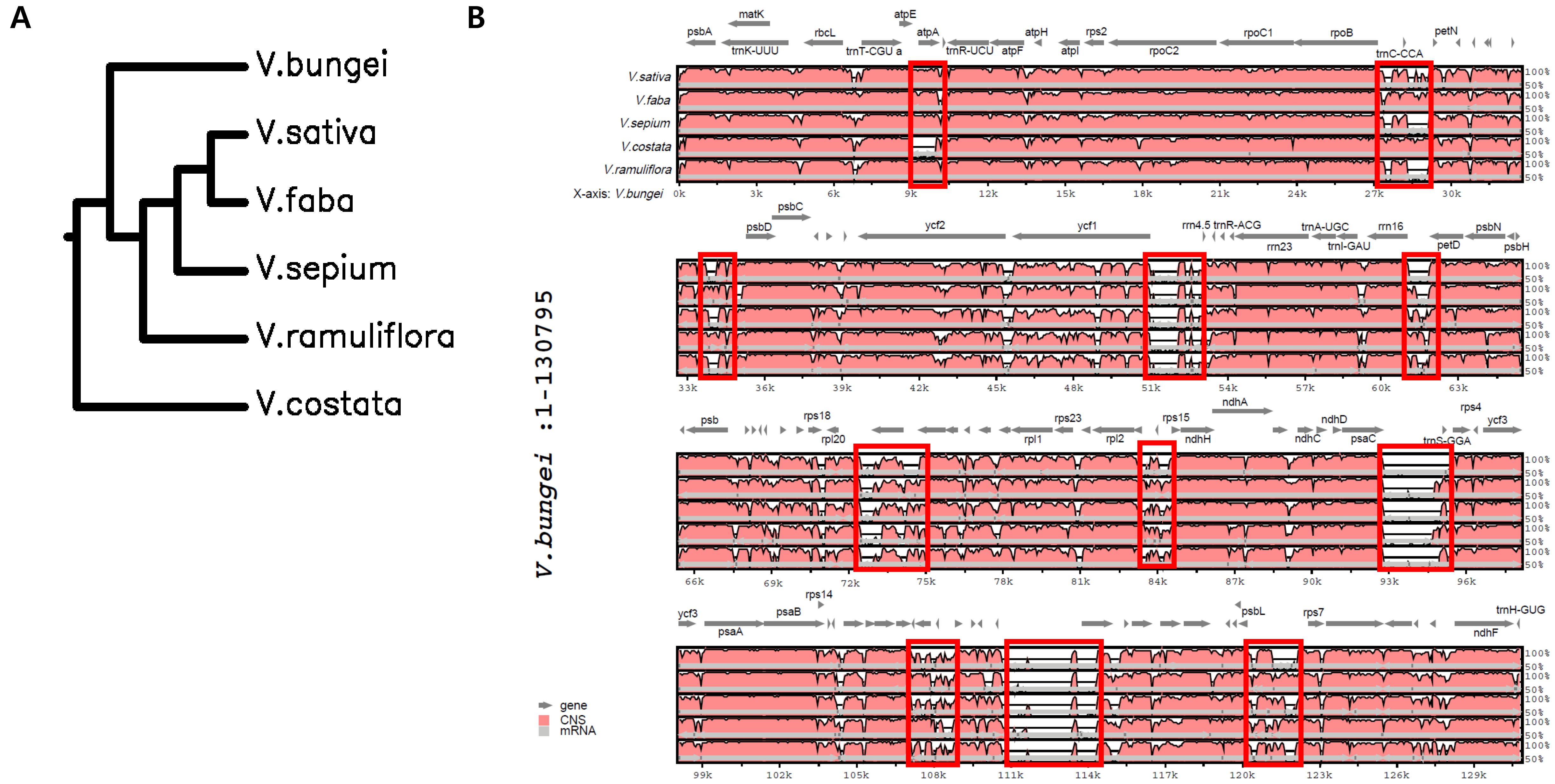

Fig. S3. Comparison of *Vicia* six species chloroplast genome. (A) phylogenetic tree based on the sequence alignment of the chloroplast genome of 6 *Vicia* species showed that *V. bungei* is far from reported other *Vicia* species. (B) Multiple alignments of chloroplast genome sequences of six *Vicia* species, each with that of *V. bungei*. Red blocks: major variant regions in chloroplast genome
